# Evaluation of the Autof MS1000 mass spectrometer in the identification of clinical isolates

**DOI:** 10.1186/s12866-020-02005-0

**Published:** 2020-10-20

**Authors:** Qiong Ma, Qi Zhang, Youhua Yuan, Wenjuan Yan, Shanmei Wang, Junhong Xu, Jiangfeng Zhang, Yuming Wang, Yi Li

**Affiliations:** 1 Department of Clinical Laboratory, Henan Provincial People’s Hospital, Henan University People’s Hospital, Zhengzhou, 450003 Henan China; 2 Henan Provincial People’s Hospital, Henan University People’s Hospital, and People’s Hospital of Henan University, Weiwu Road 5, 450003 Zhengzhou, Henan People’s Republic of China

**Keywords:** Autof MS1000, Bruker Biotyper, Mass spectrometry, Bacterial identification, Performance verification, Clinical samples

## Abstract

**Background:**

To evaluate the accuracy and performance of the Autof MS1000 mass spectrometer in bacteria and yeast identification, 2342 isolates were obtained from microbial cultures of clinical specimens (e.g. blood, cerebrospinal fluid, respiratory tract samples, lumbar puncture fluid, wound samples, stool, and urine) collected in 2019 in Henan Provincial People’s Hospital. Repetitive strains from the same patient were excluded. We tested the Autof MS1000 and Bruker Biotyper mass spectrometry systems and the classical biochemical identification system VITEK 2/API 20C AUX. Inconsistencies in strain identification among the three systems were identified by 16S rDNA and gene sequencing.

**Results:**

At the species level, the Autof MS1000 and Bruker Biotyper systems had isolate identification accuracies of 98.9 and 98.5%, respectively. At the genus level, the Autof MS1000 and Bruker Biotyper systems were 99.7 and 99.4% accurate, respectively. The instruments did not significantly differ in identification accuracy at either taxonomic level. The frequencies of unreliable identification were 1.1% (26/2342) for the Autof MS1000 and 1.5% (34/2342) for the Bruker Biotyper. In vitro experiments demonstrated that the coincidence rate of the Autof MS1000 mass spectrometer in the identification of five types of bacteria was > 93%, the identification error rate was < 3%, and the no identification rate was 0. This indicates that the Autof MS1000 system is acceptable for identification.

**Conclusions:**

The Autof MS1000 mass spectrometer can be utilised to identify clinical isolates. However, an upgradation of the database is recommended to correctly identify rare strains.

## Background

Matrix-assisted laser desorption ionisation-time of flight mass spectrometry (MALDI-TOF MS) is an emerging high-throughput technology with broad potential in clinical microbial identification because of its high resolution, speed, sensitivity, and accuracy [1–3]. Microorganism detection is based on databases of known bacteria. During detection, characteristic protein fingerprints are obtained, and these mass spectra are compared with the database for identification [4–8]. Many companies, such as Bruker Daltonics, bioMérieux, Shimadzu, Beijing Purkinje General Instrument Co., and Autobio Diagnostics, manufacture MALDI-TOF MS instruments. Recently, a new MS, the Autof MS1000 from Autobio Diagnostics, was developed for the identification of clinically important pathogenic bacteria. The Autof MS1000 has some advantages over the existing systems, such as a ion source vacuum (up to 10^− 7^ mPa) and a rapid identification module that delivers a sample result scan in 0.1 s, and can identify an entire target plate (96 isolates) in approximately 21 min. The mass spectrometer has been purchased by many laboratories in China, the United Kingdom, Italy, South Korea, and Thailand. The aim of this study was to evaluate the identification ability of the domestic Autof MS1000 in common clinical microbiology. A commercial Bruker Biotyper mass spectrometer (Bruker Daltonics, Bremen, Germany) was used as the control system. The results provide a reference for further assessment of this instrument in the medical device market.

## Results

### Isolate identification

**Fig. 1:**
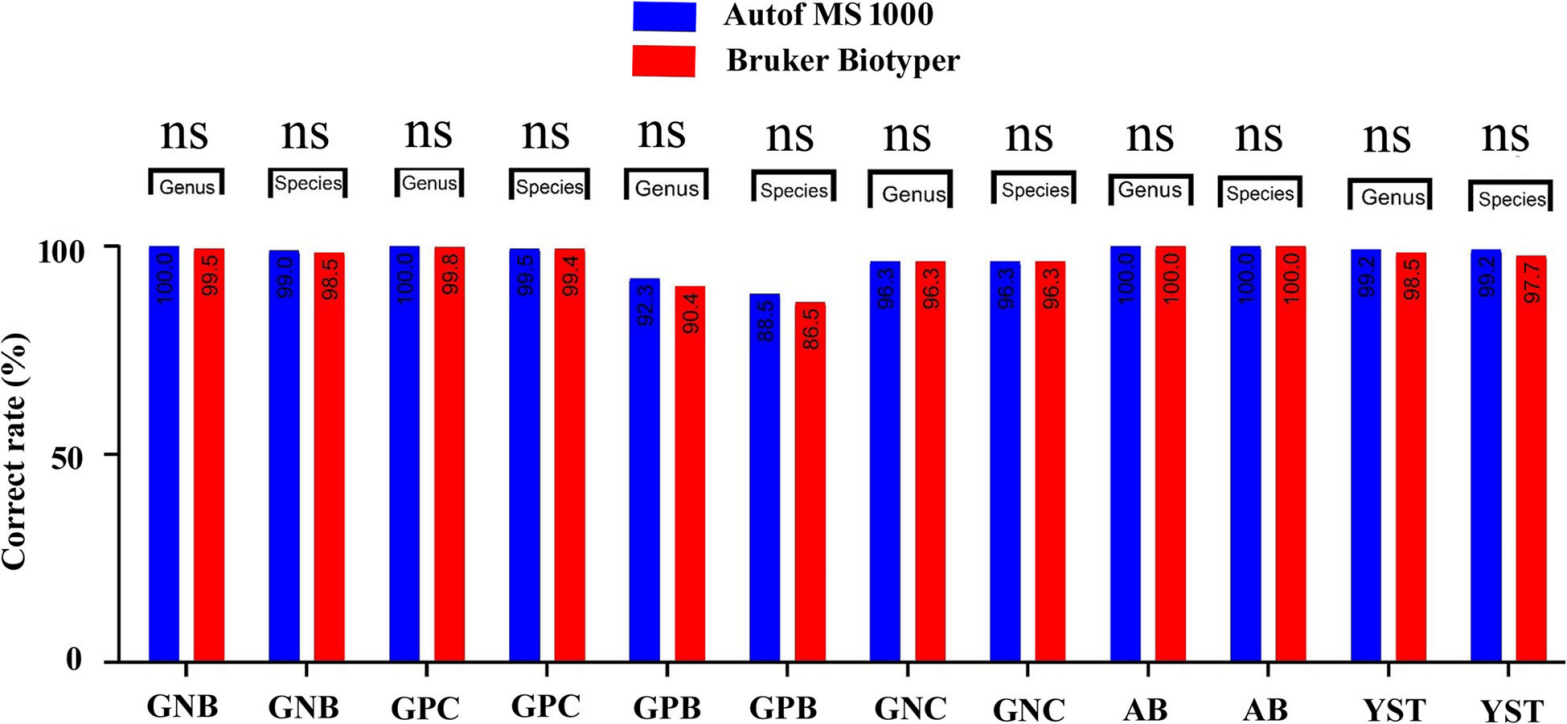
Identification results on the Autof MS1000 and Bruker Biotyper. GNB: Gram-negative bacilli; GPC: Gram-positive cocci; GPB: Gram-positive bacilli; GNC: Gram-negative cocci; AB: anaerobic bacteria; YST: yeast and yeast-like; ns: not significant

**Fig. 2:**
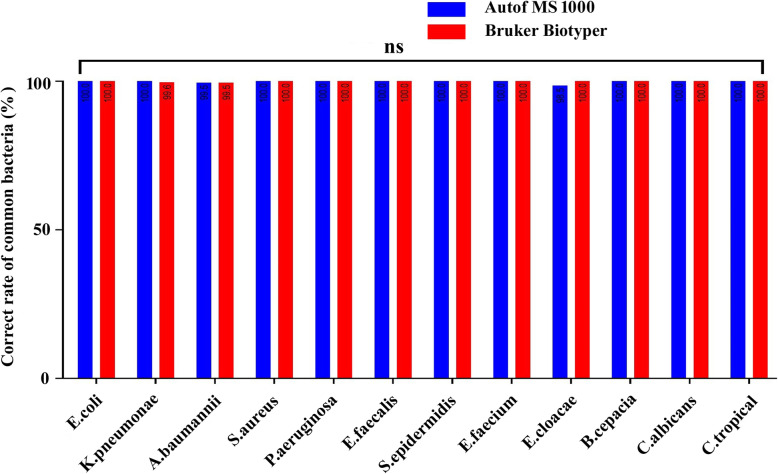
Correct rates of common bacteria and yeast on the Autof MS1000 and Bruker Biotyper. ns: not significant

There were no statistically significant differences in the identification of the 2342 strains between the two mass spectrometers at either the species or genus level. The Autof MS1000 and Bruker Biotyper systems had isolate identification accuracies of 98.9 and 98.5%, respectively, at the species level, and 99.7 and 99.4%, respectively, at the genus level. These results demonstrate that the Autof MS1000 and Bruker Biotyper mass spectrometers had equal ability to identify clinical isolates. Detailed results are shown in Fig. 1 and Additional file 1. Common bacteria and yeast were routinely obtained from microbial cultures of clinical specimens, and *Escherichia coli*, *Klebsiella pneumoniae*, *Acinetobacter baumannii*, *Staphylococcus aureus*, and *Pseudomonas aeruginosa* were most common clinical isolates. The identification accuracy for common bacteria and yeast reached 98% on the Autof MS1000. The ability to identify these isolates is of great significance to the evaluation of the MS. Detailed results are shown in Fig. 2 and Additional file 2.

### Failure rates

The Autof MS1000 incorrectly identified or failed to identify 1.1% (26/2342) of the isolates. Of these, 20 strains were identified at the genus level. The Bruker Biotyper incorrectly identified or failed to identify 1.6% (37/2342) of isolates, 21 of which were accurately identified at the genus level. The two strains of *Burkholderia pseudomallei* were identified correctly by the Autof MS1000, while the Bruker instrument failed to identify them. *B. pseudomallei* can cause melioidosis, making it an important strain with clinical significance [9, 10]. This is a major error and should be noted. The Autof instrument identified nine strains of *Salmonella* spp. and accurately identified a strain of *Salmonella enteritidis* to the species level. The Bruker instrument identified eight strains of *Salmonella* spp. and failed to identify one strain of *Salmonella paratyphi* A. Neither machine can be used for serotype identification; therefore, *Salmonella* spp. identified by mass spectrometry will require further serological typing before deciding whether to report an infectious disease. Most other identification errors were minor, such as *Citrobacter freundii* and *Raoultella planticola* being erroneously identified as *Citrobacter braakii* and *Raoultella ornithinolytica*, respectively (Table 1). Fortunately, these results will not affect clinical diagnosis or treatment decisions.

**Table 1:** Isolates misidentified at the species level or not identified by the Autof MS1000 and Bruker Biotyper

16/18S rRNA identification	*N*	Autof MS1000	Bruker Biotyper
*Acinetobacter baumannii*	1	*Acinetobacter nosocomialis*	*Acinetobacter nosocomialis*
*Enterobacter cloacae*	1	*Enterobacter cloacae/Enterobacter asburiae*	Correct identification
*Citrobacter freundii*	1	*Citrobacter freundii / Citrobacter braakii*	*Citrobacter freundii / Citrobacter braakii*
*Aeromonas hydrophila*	1	Correct identification	*Aeromonas hydrophila/Aeromonas caviae*
*Aeromonas hydrophila*	1	*Aeromonas caviae*	*Aeromonas hydrophila/Aeromonas caviae*
*Salmonella typhimurium*	4	*Salmonella* spp.	*Salmonella* spp.
*Salmonella enteritidis*	3	*Salmonella* spp.	*Salmonella* spp.
*Salmonella paratyphi A*	1	*Salmonella enterica*	No identification
*Salmonella enteritidis*	1	Correct identification	*Salmonella* spp.
*Burkholderia pseudomallei*	2	Correct identification	No identification
*Raoultella planticola*	1	*Raoultella ornithinolytica*	*Raoultella ornithinolytica*
*Raoultella planticola*	1	*Raoultella ornithinolytica*	Correct identification
*Aeromonas caviae*	1	*Aeromonas hydrophila / Aeromonas caviae*	Correct identification
*Enterobacter cancerogenus*	1	Correct identification	*Enterobacter cloacae/Enterobacter cancerogenus*
*Leifsonia shinshuensis*	1	Correct identification	*Leifsonia* spp.
*Dysgonomonas gadei*	1	Correct identification	No identification
*Staphylococcus hominis*	1	Correct identification	*Staphylococcus haemolyticus*
*Staphylococcus haemolyticus*	1	Correct identification	*Staphylococcus epidermidis*
*Staphylococcus haemolyticus*	1	*Staphylococcus hominis*	*Staphylococcus hominis*
*Nocardia asteroides*	1	Correct identification	No identification
*Nocardia otitidiscaviarum*	1	No identification	No identification
*Nocardia brasiliensis*	1	*Nocardia* spp.	*Nocardia* spp.
*Streptococcus constellatus*	2	*Streptococcus constellatus/Streptococcus anginosus*	Correct identification
*Staphylococcus gallinarum*	1	Correct identification	No identification
*Mycobacterium farcinogenes*	1	Correct identification	No identification
*Mycobacterium abscessus*	1	No identification	Correct identification
*Mycobacterium smegmatis*	1	No identification	No identification
*Actinomyces neuii*	1	Correct identification	No identification
*Streptomyces violaceoruber*	1	No identification	Correct identification
*Bacillus pumilus*	1	*Bacillus altitudinis*	*Bacillus altitudinis*
*Moraxella catarrhalis*	1	No identification	No identification
*Candida glabrata*	1	Correct identification	*Candida* spp.
*Candida rugosa*	1	No identification	No identification
*Kazachstania servazzii*	1	Correct identification	No identification

### Performance verification

**Fig. 3:**
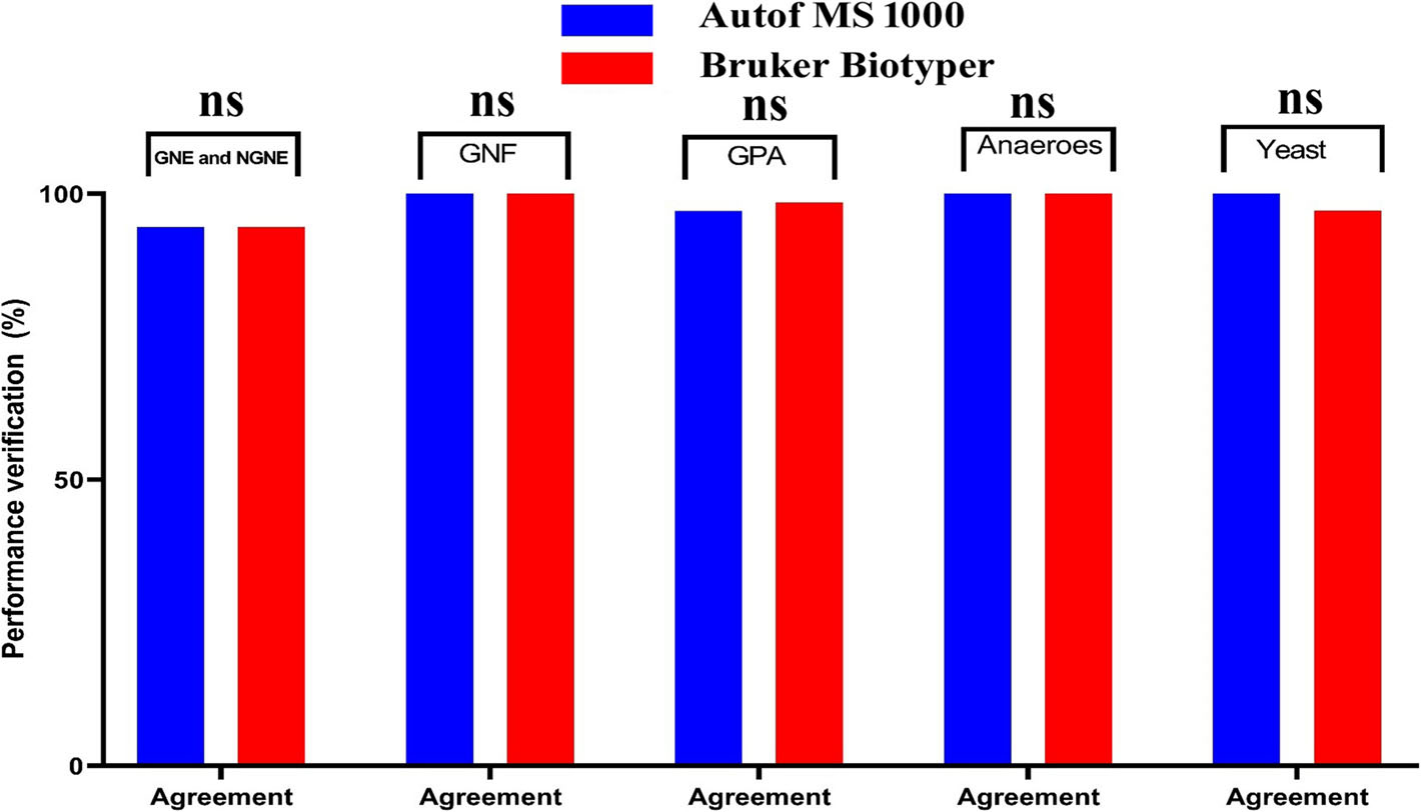
Performance verification for the Autof MS1000 and Bruker Biotyper. GNE: Gram-negative Enterobacteriaceae; NGNE: Gram-negative non-Enterobacteriaceae; GNF: Gram-negative fastidious; GPA: Gram-positive aerobic; ns: not significant

We evaluated 229 strains of Gram-negative Enterobacteriaceae, Gram-negative non-Enterobacteriaceae, Gram-negative fastidious bacteria, Gram-positive aerobic bacteria, and anaerobic bacteria, as well as yeasts and yeast-like microorganisms, according to the recommendations for the in vitro performance verification of commercial instruments in the Clinical and Laboratory Standards Institute (CLSI) M52 standard [11]. We compared the agreement, discrepancy, and unidentified isolates between the two mass spectrometers. The agreement values of both instruments were > 93%, their discrepancies were < 3, and < 2% of isolates were not identified (Fig. 3, Additional file 3). These are all acceptable values, indicating that the Autof MS1000 is a reliable system for isolate identification.

## Discussion

Bacterial identification is of great clinical significance, helping clinicians select antibiotics, accurately treat patients, and improve cure rates. To our knowledge, this is the first assessment of the identification of multiple bacteria using a Chinese mass spectrometer in central China. There were no major differences in the identification of multiple bacteria between the Chinese instrument and an imported mass spectrometer.

MALDI-TOF MS has advanced rapidly in recent years and is gradually replacing biochemical methods as the preferred tool for clinical bacterial identification [12–15]. The accuracy of MALDI-TOF MS identification depends on the collection of protein fingerprint data for all possible strains in a database [16]. The Autof MS1000 has a database of 9050 strains and 2727 species, and the Bruker Biotyper database has 5989 strains and 2371 species. Comparing the accuracy of strain identification is primarily a function of comparing strain databases; therefore, construction of the database (coverage, type, etc.) is critical [17].

For Gram-negative bacilli isolates requiring species-level identification (*n* = 1449), the analytical accuracies of the two systems were similar (99.0 and 98.7% for the Autof MS1000 and Bruker Biotyper systems, respectively (*p* = 0.490)). However, certain closely related microorganisms cannot be distinguished from one another using MALDI-TOF MS, such as *Aeromonas*, *Raoultella*, *Enterobacter*, *Acinetobacter*, and *Citrobacter* spp. Similar conclusions have been reported by several other researchers, who could not distinguish these closely related species [18–22]. Hence, for closely related species or subspecies, MALDI-TOF MS should be used in combination with biochemical and molecular methods. For *Salmonella* spp. identification, the limitations of MALDI-TOF MS must be considered [23]. Biochemical and serological tests will still be required to accurately identify *Salmonella* spp.

In this experiment, a coagulase-negative staphylococcus was isolated from blood cultures. Species-level reporting is sometimes essential to determine the clinical significance of culture isolates of coagulase-negative staphylococci [24]. The Autof MS1000 allowed better identification of *Staphylococcus hominis* and *Staphylococcus haemolyticus* than the Bruker Biotyper, suggesting that the Autof MS1000 has increased specificity for the identification of these species. However, this will require further verification with increased sample size and additional species. Viridans streptococci isolates were all correctly identified by the Bruker Biotyper, whereas two strains of *Streptococcus constellatus* were not identified to the species level by the Autof MS1000. Using the Bruker Biotyper, one strain of *Leifsonia shinshuensis*, one strain of *Staphylococcus gallinarum*, two strains of *Burkholderia pseudomallei*, and one strain of *Kazachstania servazzii* could not be identified, as these strains were not included in the Bruker Biotyper database (v5.0 5898). Database updates may resolve the difficulties in distinguishing these species.

Among the rare strains that were misidentified, *Mycobacterium* spp., *Nocardia* spp., and *Actinomyces* spp. were not correctly identified by either system. MALDI-TOF MS does have limitations in the identification of mycobacteria, *Nocardia* spp. and other aerobic actinomycetes found in the clinical microbiology laboratory [25]. This is due to the presence of multiple strains, which are not fully represented in the database. Although some strains are included, they cannot be accurately identified even with repeated operations. It may be that the protein profiles they produce are inconsistent with the characteristic profile in the database. In that case, the strain diversity of the database should be increased. Another limitation in the use of MALDI-TOF MS with slowly growing *Mycobacterium* spp. and *Actinomyces* spp. is the potential occurrence of the two species in mixed cultures, which will be recognised as the colonies on the culture plate mature, but are misidentified by MALDI-TOF MS. In addition, the sample preparation method may be an important factor for successful identification, particularly for species that are difficult to lyse, such as *Mycobacterium* spp. and *Nocardia* spp. A two-step cell disruption protocol combining the use of 0.5-mm diameter silica/zirconia beads and sonication for 15 min greatly improves the efficacy of mycobacterial identification by MALDI-TOF MS [26].

This study has some limitations. First, the sample size should be increased, and the species detected should be expanded to include more rare bacteria. Second, we did not evaluate the identification of filamentous fungi. Therefore, we will increase the sample size and analyse filamentous fungi identification in subsequent evaluations.

## Conclusions

In summary, both the Autof MS1000 and Bruker Biotyper meet the clinical requirements for bacterial identification. However, for some closely related bacteria, accurate identifications should be obtained by combining morphological, phenotypic, and molecular characteristics. A lack of diversity in database strains is also a major factor affecting the ability to identify bacteria by MALDI-TOF MS [27]. MALDI-TOF MS databases are constantly expanding, and instrument databases should be regularly updated to ensure optimal isolate identification.

## Methods

### Sample collection

A total of 2342 clinical isolates, excluding duplicate strains (172 species, 76 genera) were obtained from bacterial cultures of clinical specimens (e.g. blood, cerebrospinal fluid, respiratory tract samples, lumbar puncture fluid, wound samples, pus, ear secretion, stool, and urine) collected at Henan Provincial People’s Hospital (Zhengzhou, China) in 2019. This study was approved by the Ethics Committee of Henan Provincial People’s Hospital, Henan, China (20190050). The 2342 clinical isolates contained aerobic Gram-negative bacilli (1449 strains), aerobic Gram-negative cocci (27 strains), aerobic Gram-positive bacilli (52 strains), aerobic Gram-positive cocci (659 strains), anaerobes (48 strains), and yeasts (108 strains). Fresh samples were cultured using a variety of commonly used solid media, including tryptic soy agar with 5% sheep’s blood (BAP), chocolate agar (CHOC), and Sabouraud dextrose agar (SAB). Most specimens were incubated for 18–24 h at 36 ± 1 °C, whereas some required additional time for sufficient growth. For example, some anaerobes required up to 72 h of incubation for reliable species-level identification.

### Quality control

*E. coli* (American Type Culture Collection (ATCC)25,922), *S. aureus* (ATCC29213), *P. aeruginosa* (ATCC27853), *Enterococcus faecalis* (ATCC51299), *Enterococcus faecium* (ATCC19434), *Bacteroides fragilis* (ATCC25285), and *Candida albicans* (ATCC10231) were used as reference strains. A microorganism identification calibrator was used for the Autof MS1000 and an IVD BTS solution calibrator was used for the Bruker Biotyper. Negative controls consisted of reagents only (usually α-cyano-4-hydroxycinnamic acid matrix) and were included to detect false-positive results and reagent contamination. The Bruker Biotyper uses non-disposable target slides, and the negative control was placed at different target positions in different runs to control for location-based differences. The Autof MS1000 uses disposable target slides; therefore, the negative control was not used.

### Instruments and reagents

A Bruker Biotyper system (Bruker Daltonics, Bremen, Germany), an Autof MS1000 system (Autobio Diagnostics, Zhengzhou, China), and supporting consumables from the respective manufacturers were used. Reference strains were obtained from the ATCC (Manassas, VA, USA). Other materials, including BAP, CHOC, and SAB, were purchased from Zhengzhou Autobio Co., Ltd. (Zhengzhou, China).

### Identification using the Vitek 2 compact and API 20C AUX system

Based on colony morphology and staining results, a corresponding identification card was selected for each isolate. Identification results were automatically interpreted by the system according to the product manual, using the established algorithm. When the isolate was properly assigned to a given species or identified with low discrimination but resolved by supplemental tests, the identification was considered reliable.

### Bacterial identification by MALDI-TOF MS

MS quality control and operation were performed according to the CLSI M58 standard [28] and the Chinese Expert Consensus for Clinical Microbial Mass Spectrometry Application [29]. Deposit preparation and analysis were similar for both systems. For the Autof MS1000, protein spectra were analysed with Autof Acquirer version 1.0.55 software and library v1.1.09050. The manufacturer’s interpretation criteria were applied, with identification scores ≥9 considered positive at the species level, scores of 6–9 considered positive at the genus level, and scores < 6 defined as not identified.

On the Bruker Biotyper, extraction procedures were performed according to the product manual. Protein spectra were analysed with Bruker Biotyper 3.1 software and library v5.0 5898. The manufacturer’s interpretation criteria were applied, with identification scores ≥2.0 considered positive at the species level, scores of 1.7–2 considered positive at the genus level, and scores < 1.7 defined as not identified.

### Sequencing

For certain strains, when both mass spectrometry identifications and the biochemical identification were inconsistent at the species level, the isolate was sent to Beijing Ruiboxing Co., Ltd. (Beijing, China) for confirmation by sequencing. If the mass spectrometry identifications at the species and genus levels were inconsistent with the results of 16S rDNA sequencing, then the mass spectrometry results were considered incorrect. The 16S rRNA genes of all bacteria were sequenced, along with *dnaJ*, *sodA*, *tuf*, or *ropB* for Gram-positive cocci [30, 31]; *ropB*, *gyrB*, *recA*, or *cpn60* for Gram-negative bacteria [30, 32]; and *ropB*, *gyrB*, *SecA1*, or *hsp65* for Gram-positive bacilli [30, 33]. For yeasts, the internal transcribed spacer located between the nuclear 18S and 26S rRNA genes was sequenced [30].

### Selection principles for the performance evaluation strains

Non-reference methods were used for comparison according to the CLSI M52 standard for the verification of the in vitro performance of commercial instruments [11]. Five kinds of bacteria (Gram-negative Enterobacteriaceae, Gram-negative non-Enterobacteriaceae, Gram-negative fastidious bacteria, Gram-positive aerobic bacteria, and anaerobic bacteria), and yeast-like fungi were evaluated using three parameters: the agreement (% agreement), identification error (% discrepancy) and unidentified species (% not identified) rates. Methods with ≥93% agreement, < 3% discrepancy, and < 2% of species not identified were considered acceptable.

### Statistical analysis

Statistical analysis was performed using SPSS 20.0 statistical analysis software (*IBM Corporation, Armonk, NY, USA*). Categorical variables were compared with Chi-squared or Fisher’s exact tests. Two-tailed *p* values < 0.05 were considered statistically significant. Figures were generated using GraphPad Prism version 8.0 (GraphPad Software Inc., La Jolla, CA, USA).

## Supplementary information

**Additional file 1.** Identification results on the Autof MS1000 and Bruker Biotyper

**Additional file 2.** MS analysis of common bacteria and yeasts on the Autof MS1000 and Bruker Biotyper

**Additional file 3.** Verification of microbial identification on the Autof MS1000 and Bruker Biotyper

## Data Availability

The data sets used during the current study are available from the corresponding author on reasonable request.

## Abbreviations

MALDI-TOF MS: Matrix-assisted laser desorption ionisation-time of flight mass spectrometry
CLSI: Clinical and Laboratory Standards Institute
ATCC: American Type Culture Collection
BAP: Tryptic soy agar with 5% sheep’s blood
CHOC: Chocolate agar
SAB: Sabouraud dextrose agar
