# Supplementary material for: Evaluation of the Autof MS1000 mass spectrometer in the identification of clinical isolates
Source: BMC Microbiol. 2020 Oct 20;20:318. doi: 10.1186/s12866-020-02005-0 (PMC7576717; doi:10.1186/s12866-020-02005-0)
Supplement: Supplementary file 1 — Additional file 1. Identification results on the Autof MS1000 and Bruker Biotyper [file 12866_2020_2005_MOESM1_ESM.docx]

**Additional file 1.** Identification results on the Autof MS1000 and Bruker Biotyper

| **Classification** | **No. of isolates** | **Autof**  **MS1000** | **Bruker Biotyper** | **Statistical results** | **Autof**  **MS1000** | **Bruker Biotyper** | **Statistical results** |
| --- | --- | --- | --- | --- | --- | --- | --- |
|  |  | Genus level (%) | Genus level | *p* | species level (%) | species level (%) | *p* |
| **GNB** | 1449 | 1449 (100) | 1445 (99.7) | 0.125 | 1434 (99.0) | 1430 (98.7) | 0.490 |
| **GNC** | 27 | 26 (96.3) | 26 (96.3) | 1 | 26 (96.3) | 26 (96.3) | 1 |
| **GPC** | 659 | 659 (100) | 658 (99.8) | 1 | 656 (99.5) | 655 (99.4) | 1 |
| **GPB** | 52 | 48 (92.3) | 47 (90.4) | 1 | 46 (88.5) | 45 (86.5) | 0.767 |
| **AB** | 22 | 22 (100) | 22 (100) | 1 | 22 (100) | 22 (100) | 1 |
| **YST** | 133 | 132 (99.2) | 131 (98.5) | 1 | 132 (99.2) | 130 (97.7) | 0.622 |
| **Total** | 2342 | 2336 (99.7) | 2329 (99.4) | 0.108 | 2316 (98.9) | 2308 (98.5) | 0.299 |

Abbreviations: GNB, Gram-negative bacilli; GNC, Gram-negative cocci; GPC, Gram-positive cocci; GPB, Gram-positive bacilli; AB: anaerobic bacteria; YST: yeast and yeast-like.
