# Supplementary material for: Evaluation of the Autof MS1000 mass spectrometer in the identification of clinical isolates
Source: BMC Microbiol. 2020 Oct 20;20:318. doi: 10.1186/s12866-020-02005-0 (PMC7576717; doi:10.1186/s12866-020-02005-0)
Supplement: Supplementary file 2 — Additional file 2. MS analysis of common bacteria and yeasts on the Autof MS1000 and Bruker Biotyper [file 12866_2020_2005_MOESM2_ESM.docx]

**Additional file 2.** MS analysis of common bacteria and yeasts on the Autof MS1000 and Bruker Biotyper

| **Classification** | ***N*** | **No. (%) of strains identified with the Autof MS1000** | | | **No. (%) of strains identified with the Bruker Biotyper** | | | **Comparison** |
| --- | --- | --- | --- | --- | --- | --- | --- | --- |
|  |  | Species level (%) | Misidentified (%) | Not identified (%) | Species level (%) | Misidentified (%) | Not identified (%) | *P* |
| **GNB** |  |  |  |  |  |  |  |  |
| *Escherichia coli* | **447** | **447 (100)** | **0** | **0** | **447 (100)** | **0** | **0** | 1 |
| *Klebsiella pneumoniae* | **246** | **246 (100)** | **0** | **0** | **246 (100)** | **0** | **0** | 1 |
| *Acinetobacter baumannii* | **187** | **186 (99.5)** | **1 (0.5)** | **0** | **186 (99.5)** | **1 (0.5)** | **0** | 1 |
| *Pseudomonas aeruginosa* | **149** | **149 (100)** | **0** | **0** | **149 (100)** | **0** | **0** | 1 |
| *Enterobacter cloacae* | **65** | **64 (98.5)** | **1 (1.5)** | **0** | **65 (100)** | **0** | **0** | 1 |
| *Burkholderia cepacia* | **61** | **61 (100)** | **0** | **0** | **61 (100)** | **0** | **0** | 1 |
| *Stenotrophomonas maltophilia* | **42** | **42 (100)** | **0** | **0** | **42 (100)** | **0** | **0** | 1 |

**Additional file 2.** MS analysis of common bacteria and yeasts on the Autof MS1000 and Bruker Biotyper (continued)

| **Classification** | ***N*** | **No. (%) of strains identified with the Autof MS1000** | | | **No. (%) of strains identified with the Bruker Biotyper** | | | **Comparison** |
| --- | --- | --- | --- | --- | --- | --- | --- | --- |
|  |  | Species level (%) | Misidentified (%) | Not identified (%) | Species level (%) | Misidentified (%) | Not identified (%) | *P* |
| **GNB** |  |  |  |  |  |  |  |  |
| *Proteus mirabilis* | **38** | **38 (100)** | **0** | **0** | **38 (100)** | **0** | **0** | 1 |
| *Serratia marcescens* | **28** | **28 (100)** | **0** | **0** | **28 (100)** | **0** | **0** | 1 |
| *Klebsiella oxytoca* | **23** | **23 (100)** | **0** | **0** | **23 (100)** | **0** | **0** | 1 |
| *Haemophilus influenzae* | **20** | **20 (100)** | **0** | **0** | **20 (100)** | **0** | **0** | 1 |
| *Enterobacter aerogenes* | **16** | **16 (100)** | **0** | **0** | **16 (100)** | **0** | **0** | 1 |
| *Morganella morganii* | **13** | **13 (100)** | **0** | **0** | **13 (100)** | **0** | **0** | 1 |
| *Citrobacter freundii* | **13** | **12 (92.3)** | **1 (7.7)** | **0** | **12 (92.3)** | **1 (7.7)** | **0** | 1 |

**Additional file 2.** MS analysis of common bacteria and yeasts on the Autof MS1000 and Bruker Biotyper (continued)

| **Classification** | ***N*** | **No. (%) of strains identified with the Autof MS1000** | | | **No. (%) of strains identified with the Bruker Biotyper** | | | **Comparison** |
| --- | --- | --- | --- | --- | --- | --- | --- | --- |
|  |  | Species level (%) | Misidentified (%) | Not identified (%) | Species level (%) | Misidentified (%) | Not identified (%) | *P* |
| **GNB** |  |  |  |  |  |  |  |  |
| *Aeromonas hydrophila* | **10** | **9 (90)** | **1 (10)** | **0** | **8 (80)** | **2 (20)** | **0** | 1 |
| *Salmonella* spp. | **9** | **1 (11.1)** | **8 (88.9)** | **0** | **0** | **8 (88.9)** | **1 (11.1)** | 1 |
| *Proteus vulgaris* | **9** | **9 (100)** | **0** | **0** | **9 (100)** | **0** | **0** | 1 |
| Other | **73** | **70 (95.9)** | **3 (4.1)** | **0** | **66 (90.4)** | **4 (5.5)** | **3 (4.1)** | 0.263 |
| **Subtotal** | **1449** | **1434**  **(99.0)** | **15 (1.0)** | **0** | **1430**  **(98.7)** | **15 (1.0)** | **4 (0.3)** | 0.189 |
| **GNC** |  |  |  |  |  |  |  |  |
| *Moraxella catarrhalis* | **15** | **14 (93.3)** | **0** | **1 (6.7)** | **14 (93.3)** | **0** | **1 (6.7)** | 1 |
| Other | **12** | **12 (100)** | **0** | **0** | **12 (100)** | **0** | **0** | 1 |

**Additional file 2.** MS analysis of common bacteria and yeast on the Autof MS1000 and Bruker Biotyper (continued)

| **Classification** | ***N*** | **No. (%) of strains identified with the Autof MS1000** | | | **No. (%) of strains identified with the Bruker Biotyper** | | | **Comparison** |
| --- | --- | --- | --- | --- | --- | --- | --- | --- |
|  |  | Species level (%) | Misidentified (%) | Not identified (%) | Species level (%) | Misidentified (%) | Not identified (%) | *P* |
| **GNC** |  |  |  |  |  |  |  |  |
| **Subtotal** | **27** | **26 (96.3)** | **0** | **1 (3.7)** | **26 (96.3)** | **0** | **1 (3.7)** | 1 |
| **GPC** |  |  |  |  |  |  |  |  |
| *Staphylococcus aureus* | **175** | **175 (100)** | **0** | **0** | **175 (100)** | **0** | **0** | 1 |
| *Enterococcus faecium* | **103** | **103 (100)** | **0** | **0** | **103 (100)** | **0** | **0** | 1 |
| *Staphylococcus epidermidis* | **79** | **79 (100)** | **0** | **0** | **79 (100)** | **0** | **0** | 1 |
| *Enterococcus faecalis* | **67** | **67 (100)** | **0** | **0** | **67 (100)** | **0** | **0** | 1 |
| *Staphylococcus hominis* | **56** | **56 (100)** | **0** | **0** | **55 (98.2)** | **1 (1.8)** | **0** | 1 |
| *Streptococcus pneumoniae* | **28** | **28 (100)** | **0** | **0** | **28 (100)** | **0** | **0** | 1 |

**Additional file 2.** MS analysis of common bacteria and yeast on the Autof MS1000 and Bruker Biotyper (continued)

| **Classification** | ***N*** | **No. (%) of strains identified with the Autof MS1000** | | | **No. (%) of strains identified with the Bruker Biotyper** | | | **Comparison** |
| --- | --- | --- | --- | --- | --- | --- | --- | --- |
|  |  | Species level (%) | Misidentified (%) | Not identified (%) | Species level (%) | Misidentified (%) | Not identified (%) | *P* |
| **GPC** |  |  |  |  |  |  |  |  |
| *Staphylococcus haemolyticus* | **23** | **22 (95.7)** | **1 (4.3)** | **0** | **21 (91.3)** | **2 (8.7)** | **0** | 1 |
| *Staphylococcus capitis* | **19** | **19 (100)** | **0** | **0** | **19 (100)** | **0** | **0** | 1 |
| *Streptococcus anginosus* | **16** | **16 (100)** | **0** | **0** | **16 (100)** | **0** | **0** | 1 |
| *Streptococcus agalactiae* | **13** | **13 (100)** | **0** | **0** | **13 (100)** | **0** | **0** | 1 |
| *Streptococcus pyogenes* | **9** | **9 (100)** | **0** | **0** | **9 (100)** | **0** | **0** | 1 |
| Other | **52** | **50 (96.2)** | **2 (3.8)** | **0** | **51 (98.1)** | **0** | **1 (1.9)** | 0.495 |
| **Subtotal** | **659** | **656 (99.5)** | **3 (0.5)** | **0** | **655 (99.4)** | **3 (0.5)** | **1 (0.2)** | 1 |

**Additional file 2.** MS analysis of common bacteria and yeast on the Autof MS1000 and Bruker Biotyper (continued)

| **Classification** | ***N*** | **No. (%) of strains identified with the Autof MS1000** | | | **No. (%) of strains identified with the Bruker Biotyper** | | | **Comparison** |
| --- | --- | --- | --- | --- | --- | --- | --- | --- |
|  |  | Species level (%) | Misidentified (%) | Not identified (%) | Species level (%) | Misidentified (%) | Not identified (%) | *P* |
| **GPB** |  |  |  |  |  |  |  |  |
| *Corynebacterium striatum* | **11** | **11 (100)** | **0** | **0** | **11 (100)** | **0** | **0** | 1 |
| *Mycobacterium* spp. | **9** | **7 (77.8)** | **0** | **2 (22.2)** | **7 (77.8)** | **0** | **2 (22.2)** | 1 |
| *Nocardia* spp. | **8** | **6 (75)** | **1 (12.5)** | **1 (12.5)** | **5 (62.5)** | **1 (12.5)** | **2 (25)** | 1 |
| *Listeria monocytogenes* | **6** | **6 (100)** | **0** | **0** | **6 (100)** | **0** | **0** | 1 |
| Other | **18** | **16 (88.9)** | **1 (5.6)** | **1 (5.6)** | **16 (88.9)** | **1 (5.6)** | **1 (5.6)** | 1 |
| **Subtotal** | **52** | **46 (88.5)** | **2 (3.8)** | **4 (7.7)** | **45 (86.5)** | **2 (3.8)** | **5 (9.6)** | 1 |

**Additional file 2.** MS analysis of common bacteria and yeast on the Autof MS1000 and Bruker Biotyper (continued)

| **Classification** | ***N*** | **No. (%) of strains identified with the Autof MS1000** | | | **No. (%) of strains identified with the Bruker Biotyper** | | | **Comparison** |
| --- | --- | --- | --- | --- | --- | --- | --- | --- |
|  |  | Species level (%) | Misidentified (%) | Not identified (%) | Species level (%) | Misidentified (%) | Not identified (%) | *P* |
| **AB** |  |  |  |  |  |  |  |  |
| *Bacteroides fragilis* | **14** | **14 (100)** | **0** | **0** | **14 (100)** | **0** | **0** | 1 |
| Other | **8** | **8 (100)** | **0** | **0** | **8 (100)** | **0** | **0** | 1 |
| **Subtotal** | **22** | **22 (100)** | **0** | **0** | **22 (100)** | **0** | **0** | 1 |
| **YST** |  |  |  |  |  |  |  |  |
| *Candida albicans* | **48** | **48 (100)** | **0** | **0** | **48 (100)** | **0** | **0** | 1 |
| *Candida tropical* | **41** | **41 (100)** | **0** | **0** | **41 (100)** | **0** | **0** | 1 |
| *Candida glabrata* | **19** | **19 (100)** | **0** | **0** | **18 (94.7)** | **1 (5.3)** | **0** | 1 |
| *Candida parapsilosis* | **12** | **12 (100)** | **0** | **0** | **12 (100)** | **0** | **0** | 1 |

**Additional file 2.** MS analysis of common bacteria and yeast on the Autof MS1000 and Bruker Biotyper (continued)

| **Classification** | ***N*** | **No. (%) of strains identified with the Autof MS1000** | | | **No. (%) of strains identified with the Bruker Biotyper** | | | **Comparison** |
| --- | --- | --- | --- | --- | --- | --- | --- | --- |
|  |  | Species level (%) | Misidentified (%) | Not identified (%) | Species level (%) | Misidentified (%) | Not identified (%) | *P* |
| **YST** |  |  |  |  |  |  |  |  |
| Other | **13** | **12 (92.3)** | **0** | **1 (7.7)** | **11 (84.6)** | **0** | **2 (15.4)** | 1 |
| **Subtotal** | **133** | **132 (99.2)** | **0** | **1 (0.8)** | **130 (97.7)** | **1 (0.8)** | **2 (1.5)** | 0.622 |
| **Total** | **2342** | **2316 (98.9)** | **20 (0.9)** | **6 (0.3)** | **2308 (98.5)** | **21 (0.9)** | **13 (0.6)** | 0.270 |

Abbreviations: GNB: Gram-negative bacilli; GNC: Gram-negative cocci; GPB: Gram-positive bacilli; GPC: Gram-positive cocci; AB: anaerobic bacteria; YST: yeast and yeast-like.
