# Supplementary material for: Evaluation of the Autof MS1000 mass spectrometer in the identification of clinical isolates
Source: BMC Microbiol. 2020 Oct 20;20:318. doi: 10.1186/s12866-020-02005-0 (PMC7576717; doi:10.1186/s12866-020-02005-0)
Supplement: Supplementary file 3 — Additional file 3. Verification of microbial identification on the Autof MS1000 and Bruker Biotyper [file 12866_2020_2005_MOESM3_ESM.docx]

**Additional file 3.** Verification of microbial identification on the Autof MS1000 and Bruker Biotyper

| **Organism Type** | ***N*** | **Autof MS1000** | | | **Bruker Biotyper** | | | **Statistical results** | |
| --- | --- | --- | --- | --- | --- | --- | --- | --- | --- |
|  |  | Agreement % | No identification % | Discrepancy % | Agreement % | No identification % | Discrepancy % | *X^2^* | *P* |
| Gram-negative Enterobacteriaceae and Non-Enterobacteriaceae | 69 | 94.2 | 0 | 2.9 | 94.2 | 0 | 2.9 | 0 | 1 |
| Gram-negative fastidious | 30 | 100 | 0 | 0 | 100 | 0 | 0 | - | 1 |
| Gram-positive aerobic | 66 | 97.0 | 0 | 3.0 | 98.5 | 0 | 1.5 | 0 | 1 |
| Anaerobic | 30 | 100 | 0 | 0 | 100 | 0 | 0 | 0 | 1 |
| Yeast and yeast-like | 34 | 100 | 0 | 0 | 97.1 | 0 | 2.9 | - | 1 |
| Total | 229 | 97.4 | 0 | 2.6 | 97.4 | 0 | 2.6 | 0 | 1 |

-：Fisher test without *X*^2^ value.
